# Neoadjuvant chemotherapy for patients with international federation of gynecology and obstetrics stages IB3 and IIA2 cervical cancer: a multicenter prospective trial

**DOI:** 10.1186/s12885-022-10355-3

**Published:** 2022-12-05

**Authors:** Yingjie Hu, Yingyan Han, Yuanming Shen, Jing Chen, Yaheng Chen, Yile Chen, Junying Tang, Min Xue, Li Hong, Wenjun Cheng, Danbo Wang, Zhiqing Liang, Yifeng Wang, Qinghua Zhang, Hui Xing, Yu Zhang, Cunjian Yi, Zhiying Yu, Youguo Chen, Manhua Cui, Cailing Ma, Hongying Yang, Ruizhen Li, Ping Long, Yu Zhao, Pengpeng Qu, Guangshi Tao, Lihua Yang, Sufang Wu, Zhihua Liu, Ping Yang, Weiguo Lv, Xing Xie, Ding Ma, Hui Wang, Kezhen Li

**Affiliations:** 1 Department of Gynecology and Obstetrics, Tongji Hospital, Tongji Medical College, Huazhong University of Science and Technology, Wuhan, Hubei China; 2 Department of Gynecologic Oncology, Women’s Hospital, School of Medicine, Zhejiang University, Hangzhou, Zhejiang China; 3 Department of Gynecologic Oncology, Hunan Cancer Hospital/The Affiliated Cancer Hospital of Xiangya School of Medicine, Central South University, Changsha, Hunan China; 4 Department of Gynecology, The First Affiliated Hospital of Chongqing Medical University, Chongqing, China; 5 Department of Gynecology and Obstetrics, The Third Xiangya Hospital of Central South University, Changsha, Hunan China; 6 Department of Obstetrics and Gynaecology, Renmin Hospital of Wuhan University, Wuhan, Hubei China; 7 Department of Gynecology, the First Affiliated Hospital of Nanjing Medical University, Nanjing, Jiangsu China; 8 Department of Gynecology, Cancer Hospital of China Medical University, Liaoning Cancer Hospital and Institute, Shenyang, Liaoning China; 9 Department of Obstetrics and Gynecology, Southwest Hospital, Army Medical University (Third Military Medical University), Chongqing, China; 10 Department of Gynecology, Obstetrics and Gynecology Center, Zhujiang Hospital, Southern Medical University, Guangzhou, Guangdong China; 11 Department of Obstetrics and Gynecology, The Central Hospital of Wuhan, Tongji Medical College, Huazhong University of Science and Technology, Wuhan, Hubei China; 12 Department of Obstetrics and Gynaecology, Xiangyang Central Hospital, Affiliated Hospital of Hubei University of Arts and Science, Xiangyang, Hubei China; 13 Department of Gynecology, Xiangya Hospital, Central South University, Changsha, Hunan China; 14 Department of Obstetrics and Gynecology, The First Affiliated Hospital of Yangtze University, Jingzhou, Hubei China; 15 Department of Gynecology, The First Affiliated Hospital of Shenzhen University, Health Science Center; Shenzhen Second People’s Hospital, Shenzhen, Guangdong China; 16 Department of Obstetrics and Gynecology, The First Affiliated Hospital of Soochow University, Suzhou, Jiangsu China; 17 Department of Gynecology and Obstetrics, The Second Hospital of Jilin University, Changchun, Jilin China; 18 Department of Gynecology, the First Affiliated Hospital of Xinjiang Medical University, Urumqi, Xinjiang China; 19 Department of Gynecology, Yunnan Tumor Hospital and The Third Affiliated Hospital of Kunming Medical University, Kunming, Yunnan China; 20 Department of Gynecology and Obstetrics, Shenzhen Hospital of Beijing University, Shenzhen, Guangdong China; 21 The Second People’s Hospital of Jingmen, Hubei Jingmen, China; 22 Department of Obstetrics and Gynecology, The Second Affiliated Hospital of Wenzhou Medical University, Wenzhou, Zhejiang China; 23 Department of Gynecology Oncology, Tianjin Central Hospital of Gynecology and Obstetrics, Tianjin, China; 24 Department of Obstetrics and Gynecology, The Second Xiangya Hospital of Central South University, Changsha, Hunan China; 25 Department of Obstetrics and Gynecology, The Second Affiliated Hospital, Kunming Medical University, Kunming, Yunnan China; 26 Department of Obstetrics and Gynecology, Shanghai General Hospital, Shanghai Jiao Tong University School of Medicine, Shanghai, China; 27 Department of Gynecology, Affiliated Shenzhen Maternity and Child Healthcare Hospital, Southern Medical University, Shenzhen, Guangdong China; 28 Department of Obstetrics and Gynecology, First Affiliated Hospital, School of Medicine, Shihezi University, Shihezi, Xinjiang China

**Keywords:** Cervical cancer, Neoadjuvant chemotherapy, Radical surgery, Prognosis, Non-responders

## Abstract

**Background:**

Preoperative neoadjuvant chemotherapy (NACT) has been widely used in developing countries for the treatment of patients with International Federation of Gynecology and Obstetrics (FIGO) stages IB3 and IIA2 cervical cancer. However, the effectiveness of NACT and treatment options for NACT-insensitive patients have been concerning. This study will assess prognostic differences between NACT and primary surgery treatment (PST), determine factors associated with prognosis, and explore better adjuvant treatment modalities for NACT-insensitive patients.

**Methods:**

This study analyzed clinical characteristics, pathological characteristics, treatment options, and follow-up information of 774 patients with FIGO stages IB3 and IIA2 cervical cancer from 28 centers from January 2016 to October 2019 who participated in a multicenter, prospective, randomized controlled trial.

**Results:**

For patients undergoing NACT, the 5-year OS and PFS rate was 85.8 and 80.5% respectively. They were similar in the PST group. There was no significant difference in OS and PFS between clinical response (CR)/partial response (PR) groups and stable disease (SD)/progressive disease (PD) groups. Apart from deep cervical invasion (*p* = 0.046) affecting OS for patients undergoing NACT, no other clinical and pathological factors were associated with OS. 97.8% of NACT-insensitive patients opted for surgery. If these patients did not have intermediate- or high-risk factors, whether they had undergone postoperative adjuvant therapy was irrelevant to their prognosis, whereas for patients with intermediate- or high-risk factors, adjuvant chemotherapy resulted in better PFS (chemotherapy vs. no therapy, *p* < 0.001; chemotherapy vs. radiotherapy, *p* = 0.019) and OS (chemotherapy vs. no therapy, *p* < 0.001; chemotherapy vs. radiotherapy, *p* = 0.002).

**Conclusions:**

NACT could be a choice for patients with FIGO stages IB3 and IIA2 cervical cancer. The main risk factor influencing prognosis in the NACT group is deep cervical invasion. After systematic treatment, insensitivity to NACT does not indicate a poorer prognosis. For NACT-insensitive patients, Chinese prefer surgery. Postoperative adjuvant therapy in patients with no intermediate- or high-risk factors does not improve prognosis, and chemotherapy in patients with intermediate- and high-risk factors is more effective than radiation therapy and other treatments.

**Trial registration:**

The study was prospectively registered on ClinicalTrials.gov (NCT03308591); date of registration: 12/10/2017.

**Supplementary Information:**

The online version contains supplementary material available at 10.1186/s12885-022-10355-3.

## Background

Cervical cancer is a major global public health problem, with an estimated 604,000 new cases and 342,000 deaths reported in 2020 [1]. The situation is even worse in developing countries, where regional morbidity is 3–10 times higher than that in developed countries [2, 3]. Concurrent platinum-based chemoradiation (CCRT) is the recommended treatment according to the National Comprehensive Cancer Network guidelines for cervical cancer, except for FIGO stages IA, IB1, IB2, and IIA1 cancer [4]. However, this is limited in clinical application in many developing countries due to inadequate radiotherapy facilities. The incidence of cervical cancer in women aged 20–29 years increased annually by 10.3% between 2000 and 2009 [5]. This is especially concerning as CCRT has a significant impact on the reproductive organs of young patients [6]. CCRT may cause premature ovarian failure, vaginal injury, pelvic tissue degeneration, and unparalleled damage to a woman’s reproductive endocrine function and sexual lifestyle [7, 8]. Patients who achieve poor outcomes after CCRT have great difficulty in undergoing reoperation to achieve tumor reduction due to severe pelvic tissue injuries. Therefore, more obstetricians and gynecologists have been recognizing the need for breakthroughs in surgical procedures and improvements in current surgical techniques, especially in developing countries.

Although physicians have attempted radical surgery (RS), patients with stage IB3 and IIA2 cancer often have huge lesions that are difficult to remove completely by surgery alone. NACT for FIGO stages IB3 and IIA2 cervical cancer is considered by some researchers to reduce the difficulty of operation. Additionally, it can improve the rate of radical resection, reduce the vaginal injury of radical radiotherapy, and lead to better outcomes in quality of life and ovarian function for premenopausal patients [9–11]. At the beginning of this century, NACT followed by RS was gradually adopted in some regions such as Europe, Asia, and South America. Eventually, it became widely used, not only increasing complete resection rates by surgery but also improving the survival of patients, on par with simultaneous chemoradiotherapy in most literature reports [12]. Nevertheless, it is controversial whether NACT significantly improves the prognosis of patients. Additionally, its effects on weakening patients’ general condition and surgical tolerance and increasing patients’ chemotherapy resistance rate are unclear. This is especially true for cases that are insensitive to NACT, in which alternative follow-up treatments and outcomes as a result of the timing of delayed treatment must be determined. Therefore, the use of radical hysterectomy after platinum-based NACT as an alternative treatment option for cervical cancer remains controversial, especially for patients with FIGO stage IB3 and IIA2 disease.

In recent years, a large number of studies on NACT in cervical cancer have been published, many of which focused on the differences between CCRT and NACT. Among them, one study from Europe (EORTC 55994) [13] and one from Asia (NCT00193739) [14] showed that NACT followed by radical hysterectomy group had similar 5-year overall survival (OS) and less delayed toxicities compared to the CCRT group. However, the best way to assess what chemotherapy adds to surgery is to compare it directly with surgery alone [15]. There are some studies focusing on the prognosis between NACT + RS and primary surgery treatment (PST), but the conclusions of these clinical trials on whether NACT plus radical hysterectomy can improve the prognosis of patients compared with primary radical hysterectomy are inconsistent [16–22]. The effectiveness of NACT and principles of postoperative treatment are the two most controversial aspects, especially for patients insensitive to NACT. Existing studies suggest that the prognosis of NACT-insensitive patients may be worse than that of NACT-sensitive patients [14, 23]. Concurrently, there is a lack of studies that specifically analyze what follow-up treatment should be administered to patients who are insensitive to NACT. In the context of advances in surgical techniques and postoperative protocols, high-quality randomized controlled trials are needed.

In this study, the clinical characteristics, pathological characteristics, and therapeutic methods of NACT of patients with FIGO stages IB3 and IIA2 cervical cancer from 28 centers in China were reviewed and the risk factors were evaluated, identifying prognostic factors, investigating treatment options, and analyzing the impact of different treatments on the prognosis of patients receiving NACT, especially for those insensitive to NACT.

## Methods

### Study Design and Participants

We analyzed the records of 774 patients with FIGO stages IB3 and IIA2 cervical cancer who participated in a multicenter, prospective, randomized controlled trial conducted at 28 hospitals in China (ClinicalTrials.gov identifier: NCT03308591). Patients were eligible if they were aged between 18 and 65 years; had a pathological diagnosis of proven invasive squamous cell carcinoma, adenocarcinoma or squamous adenocarcinoma of the uterine cervix before any treatment; and had KARNOFSKY scores ≥60. Patients were excluded from the study if they had (1) other tumors, (2) received chemotherapy or radiotherapy prior to the study, (3) other contraindications to surgery, radiotherapy, or chemotherapy, (4) any renal, hepatic, respiratory, cardiac, or mental disorders.

This trial was approved by the Ethics Committee of Tongji Hospital, Tongji Medical College, Huazhong University of Science and Technology (IRB ID: TJ-C20151201). This trial was conducted in accordance with applicable regulatory requirements and the principles of the Declaration of Helsinki. All patients received an explanation of the study aims and provided signed informed consent prior to participating.

According to postoperative pathological risk factors, patients were placed into either a high-risk group, which included patients with positive nodes, positive margins, or positive parametria; the remaining patients were further divided into a low-risk group and intermediate-risk group according to the Sedlis criteria [24]. Intermediate risk was defined as a tumor with positive lymphovascular space invasion with deep 1/3 stromal invasion, middle 1/3 stromal invasion and tumor diameter 2 cm, or superficial 1/3 stromal invasion and tumor diameter ≥ 5 cm or a tumor with no lymphovascular space invasion but with deep or middle 1/3 stromal invasion and tumor diameter ≥ 4 cm. The low-risk group included patients with negative nodes, negative margins, negative parametria, and no cervical intermediate risk factors after radical hysterectomy.

According to postoperative adjuvant therapy, patients were classified into three groups—no therapy; chemotherapy; and radiotherapy. The no therapy group included patients who did not receive any adjuvant therapy, chemotherapy group included patients only receiving adjuvant chemotherapy; and radiotherapy group included rest patients who had received at least adjuvant radiotherapy.

### Randomization

After providing signed informed consent, patients were randomly divided into the NACT and PST groups using a computer-generated random number code at the National Clinical Research Center for Gynecology and Obstetrics, Tongji Hospital, Tongji Medical College, Huazhong University of Science and Technology. Details of group allocations were maintained in sequentially numbered, opaque, sealed envelopes prepared by a statistician with no clinical involvement in the trial. Patients meeting the inclusion and exclusion criteria were randomized. A clinical research coordinator opened the envelope, assigned the patients to interventions, and informed the investigators at each center.

### Therapeutic Process

NACT or adjuvant chemotherapy included cisplatin 70–85 mg/m^2^ and paclitaxel 165–175 mg/m^2^.

Patients in the NACT group were assigned to receive two courses of platinum-based chemotherapy before surgery. Doctors appraised the effect of the chemotherapy 2–3 weeks after NACT. Responders in the NACT group underwent RS 3 weeks after NACT. The following surgical pathology indicators were evaluated: (1) lymph node metastasis, (2) parametrial infiltration, (3) ≥2/3 depth of interstitial infiltration, (4) moderately to poorly differentiated histopathology (grades 2–3), and (5) lymphovascular space invasion. Patients with any of the above risk factors received four courses of adjuvant chemotherapy, those without any of the risk factors received two courses of chemotherapy, and those who were found to have positive para-aortic lymph nodes or three or more positive pelvic lymph nodes received six courses of postoperative chemotherapy. The interval between every course was 3 weeks. Patients with positive vaginal margins required additional radiation therapy.

The follow-up treatment regimen for non-responders to NACT was either concurrent chemoradiotherapy or radical hysterectomy, determined by physicians according to their specific conditions.

Patients in the PST group underwent radical hysterectomy and pelvic lymph node dissection directly. Their postoperative adjuvant chemotherapy was based on the same risk factors as those for the NACT group. Patients with any risk factors received six courses of adjuvant chemotherapy, those without any of the risk factors received four courses of chemotherapy, and those with positive para-aortic lymph nodes or three or more positive pelvic lymph nodes received eight courses of postoperative chemotherapy. Similarly, the interval between every course was 3 weeks, and patients with positive vaginal margins required additional radiation therapy.

After treatment completion, patients were followed up every 3 months during the first 2 years and every 6 months thereafter, and the follow-up duration was from the date of entering this study to the date of death or February 2022.

### Responses to NACT

Sensitivity to NACT was evaluated using to the Response Evaluation Criteria in Solid Tumors (RECIST version 1.1) [24] according to the tumor size measured at initial diagnosis and immediately before surgery. This is widely accepted as a standard method in assessing the activity and efficacy of therapeutics in the field of solid tumor research and among cervical cancer. Sensitivity to NACT were defined as clinical response (CR) + partial response (PR), and insensitivity to NACT was defined as stable disease (SD) + progressive disease (PD). Pathological CR (pCR) was defined as the absence of tumor cells in the surgical specimen after neoadjuvant therapy.

### Statistical analysis

On the basis of previous reports [22, 25], we assumed that the 5-year disease-free survival rate (DFS) was 94% in NACT group and 80% in PST group. With an enrolment period of 2 years and a follow-up period of 1 year, and taking into account the 20% dropout rate, a total of 800 cases were planned to be enrolled to achieve 80% power and a two-sided 5% significance-level hazard ratio.

SPSS 26.0 (SPSS, Chicago, IL, United States) was used for statistical analysis. Continuous variables were compared with parametric methods if a normal distribution was confirmed. Non-normally distributed variables and categorical data were compared with nonparametric tests. The chi-squared test was used for categorical data. Cox proportional hazards regression models were employed to estimate the relative likelihood of progression-free survival (PFS) and OS with various factors in both univariate and multivariate analyses. Candidate variables with a *p*-value of < 0.2 on univariate analysis were included in the multivariable model and no adjustments were made to account for missing data. The results are presented as odds ratios (ORs) with 95% confidence intervals (CIs). Survival curves were generated using the Kaplan–Meier method and proportional hazards models were used to estimate the hazard ratios (HRs) and 95% CIs for the effect of treatment on progression-free and overall survival. To determine the therapeutic risk factor of survival outcomes, groups were compared using the log-rank test. Unless otherwise stated, all analyses were performed with a two-sided significance at a level of 0.05.

In both univariate and multivariate analyses, age, hemoglobin concentration, platelet concentration, body mass index (BMI), change in tumor size, tumor size before NACT, and tumor size after NACT were regarded as distributed variables, while the approach of surgery, FIGO stage, pathological type, lymph node metastasis, uterus involvement, vagina involvement, parametrial infiltration, lymphovascular space invasion, deep cervical invasion, and adjuvant therapy were regarded as categorical variables. The pathological type was regarded as an unordered categorical variable containing three values—squamous cell carcinoma, adenocarcinoma, or squamous adenocarcinoma.

Additional explanatory notes were added when two or more methods were used to measure the tumor size in the greatest dimension. The order of inclusion for statistics was magnetic resonance imaging (MRI), computed tomography (CT), ultrasonography, and pelvic examination (PV). For patients undergoing NACT, the preferred method of measuring tumor size after NACT remained the same as that before NACT, and the change in tumor size was calculated as tumor size before NACT minus that after NACT. The approach to surgery included minimally invasive surgery (laparoscopic and robot-assisted radical hysterectomy) and open surgery (abdominal radical hysterectomy).

## Results

### Efficacy of NACT

**Fig. 1:**
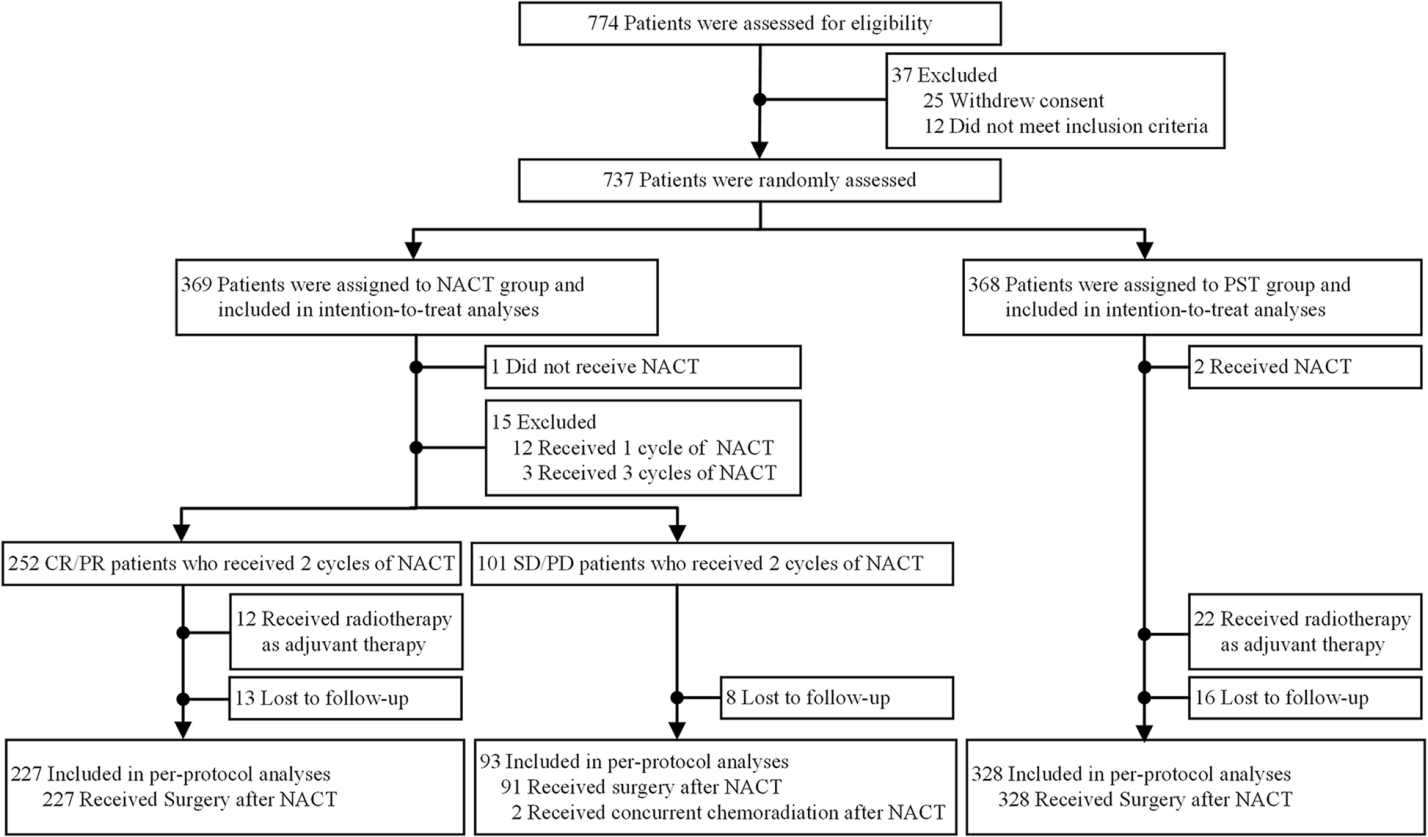
Flow Diagram of Patient Selection. Abbreviations: NACT, neoadjuvant therapy; PST, primary surgical treatment; CR, clinical response; PR, partial response; SD, stable disease; PD, progressive disease

The study was based on data from 774 patients with cervical cancer enrolled from 28 hospitals in China between January 2016 and October 2019. Consent was withdrawn by 25 patients and 12 patients did not meet the inclusion criteria. Therefore, 369 and 368 patients were randomly assigned to the NACT and PST groups, respectively. 320 patients in the NACT group and 328 patients in PST group were included in per-protocol analysis (Fig. 1). The number of patients lost to follow-up was 21 (5.9%) in the NACT group and 16 (4.3%) in the PST group.

To give a clearer view of the efficiency of NACT, we compared the baseline characteristics of the NACT and PST groups. Although the patients were randomly assigned to the two groups, the baseline demographics and clinical characteristics were not well balanced between the two groups in terms FIGO stage and tumor size (Additional file 1: Table S1). The NACT group presented a higher rate (62.6%) of patients with carcinoma invasion beyond the uterus than the PST group (44.6%, *p <* 0.001). Tumor size was also larger in the NACT group than in the PST group (*p =* 0.005), indicating that patients in the NACT group had more advanced tumors. 71.4% (252/353) patients achieved CR/PR, including 30 patients achieving pCR.

**Fig. 2:**
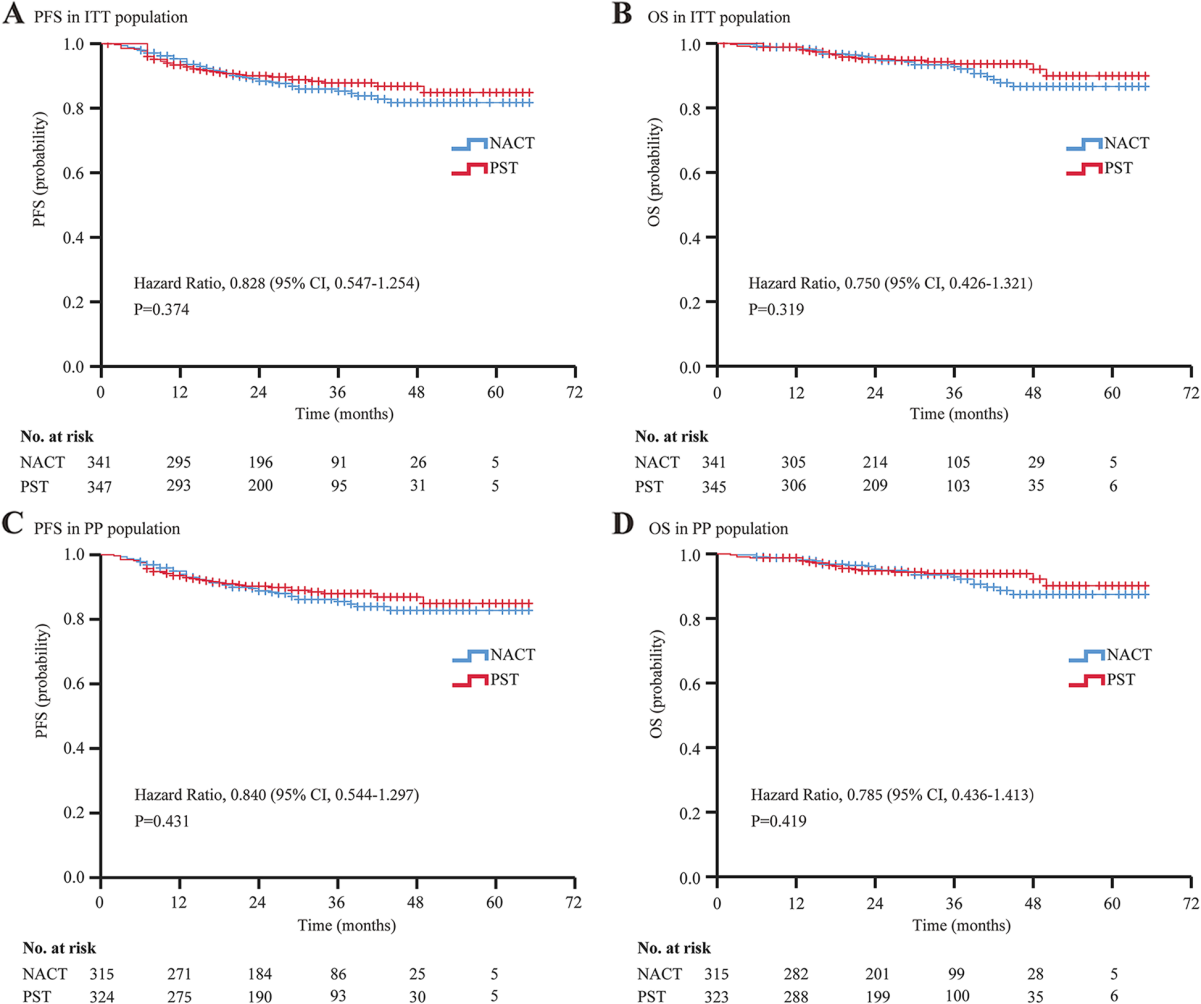
Progression Free Survival and Overall Survival Curves in Intention-to-Treat and Per-Protocol Populations. Kaplan–Meier plot for (**A**) progression free survival and (**B**) overall survival compared between NACT and PST in ITT population, and (**C**) PFS and (**D**) OS in PP population. The hazard ratio, 95% confidence interval, and corresponding *p*-value were estimated by Cox proportional-hazards models. Tick marks indicate censored data. Abbreviations: NACT, neoadjuvant therapy; PST, primary surgical treatment; PFS, progression free survival; OS, overall survival; ITT, intention-to-treat; PP, per-protocol

By February 2022, 73 months had passed since the first patient was enrolled, and the median (interquartile range [IQR]) follow-up time was 34 (26-43) months. In the Kaplan–Meier analysis, patients in the NACT group showed a similar rate of PFS to those in the PST group (HR 0.828; 95% CI, 0.574–1.254; *p =* 0.320; 5-year PFS, 80.5% vs. 83.8%). Similarly, OS in the NACT group was no worse than in the PST group (HR 0.750; 95% CI, 0.426–1.321; *P =* 0.319; 5-year OS, 85.8% vs. 87.8%) (Fig. 2).

### Characteristics and Survival Outcomes of Patients Undergoing NACT

We further performed a subgroup analysis of the NACT group based on NACT sensitivity; 227 (70.9%) patients were in CR/PR group, and 93 (29.1%) patients were in SD/PD group. No significant differences between the two groups were found with respect to the baseline characteristics accessed before receiving NACT (Additional file 1: Table S2). The median age of the patients was 48 years (range: 20–64 years), and the median BMI was 22.8 kg/m^2^ (range: 15.8–34.5 kg/m^2^). Additionally, there were no significant differences between laboratory examination results between the two groups including serum hemoglobin concentration and platelet concentration. The distribution of FIGO stage and pathological type was well balanced. All responders and 91 of 93 non-responders received RS after NACT, and all these patients had completed tumor pathology. The pathological classification was identified as follows: squamous cell carcinoma (288 cases, 90.6%), adenocarcinoma (22 cases, 6.9%), and squamous adenocarcinoma (eight cases, 2.5%). There were no significant differences between the CR/PR and SD/PD groups with respect to histological subtype—deep cervical invasion (33.9 and 44.0%, respectively), lymphovascular space invasion (12.3 and 15.4%, respectively), parametrial involvement (0.9 and 3.3%, respectively), vagina involvement (1.8 and 3.3%, respectively), uterus involvement (4.8 and 7.7%, respectively), and lymph node metastasis (10.6 and 15.4%, respectively). Only one case in the CR/PR group had a positive vaginal resection margin (Additional file 1: Table S3).

**Fig. 3:**
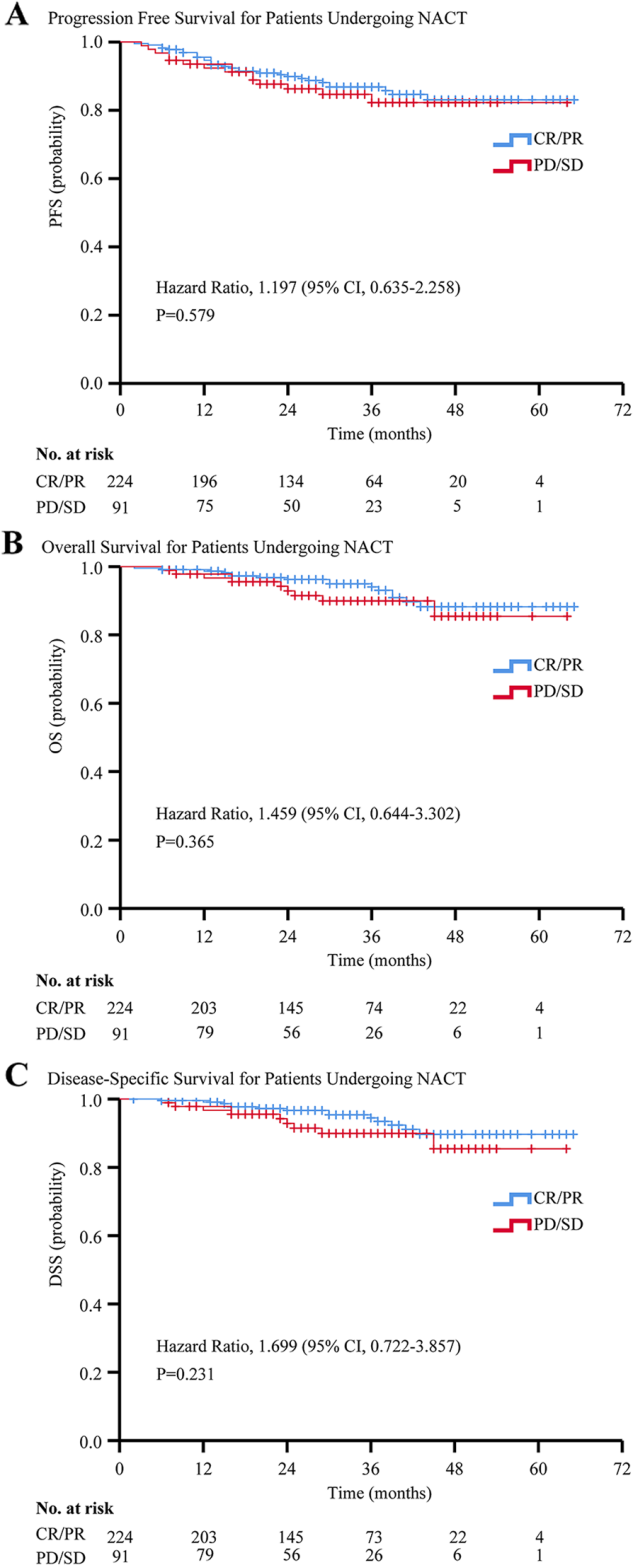
Progression Free Survival, Overall Survival, and Disease-Specific Survival Curves for Patients Undergoing NACT. Kaplan–Meier plot for (**A**) progression free survival, (**B**) overall survival, and (**C**) disease-specific survival according to the response of cervical cancer patients to chemotherapy. Tick marks indicate censored data. Abbreviations: PFS, progression-free survival; OS, overall survival; DSS, disease-specific survival; CR, clinical response; PR, partial response; SD, stable disease; PD, progressive disease

For all patients undergoing NACT, the 5-year OS rate was 86.5% (86.9% for responders and 85.5% for non-responders), and the 5-year PFS rate was 81.4% (82.1% for responders and 79.9% for non-responders). By February 2022, 44 of 320 (13.8%) patients in the NACT group experienced disease progression or death from any cause. Among them, 25 of 227 (11.0%) patients in the PR/CR group, and 13 of 93 (14.0%) patients in the SD/PD group developed recurrences. The PR/CR group had fewer distant metastases than the SD/PD group (four cases, 1.8% vs. three cases, 3.2%, respectively). Moreover, a total of 25 deaths had been reported, with 16 (7.0%) patient deaths in the PR/CR group, and nine (9.7%) patient deaths in the SD/PD group (Additional file 1: Table S4). No significant difference was found in PFS and OS between responders and non-responders (PFS: HR, 1.197 [95% CI, 0.635–2.258], *p =* 0.579; OS: HR, 1.459 [95% CI, 0.644–3.302], *p =* 0.365, Fig. 3A and B). In addition, two deaths unrelated to cancer had been reported. Specifically, one patient died of COVID-19 and one committed suicide. These two patients belonged to the PR/CR group. Therefore, we plotted the survival curves of disease-specific survival (DSS) and found that non-responders did not have poorer survival outcomes (DSS: HR, 1.699 [95% CI, 0.722–3.857], *p =* 0.231, Fig. 3C).

For 30 patients achieving pCR, one lost to follow-up, one died in COVID-19, and the other 28 survived to the last follow-up.

### Prognostic Significance of Clinicopathological Factors for the SD/PD group

To further analyze the clinicopathological risk factors affecting the prognosis of patients with locally advanced cervical cancer in the NACT group and its subgroups (CR/PR and SD/PD groups), we performed univariate analysis. As shown (Additional file 1: Table S5), for all patients undergoing NACT, lower hemoglobin concentration (Crude OR, 0.980 [95% CI, 0.966–0.994], *p =* 0.005), larger tumor size before NACT (Crude OR, 1.253 [95% CI, 1.011–1.552], *p* = 0.039) or after NACT (Crude OR, 1.255 [95% CI, 1.064–1.481], *p =* 0.007), > 1/2 depth of cervical invasion (Crude OR, 3.359 [95% CI, 1.808–6.243], *p <* 0.001), and lymph node metastasis (Crude OR, 2.350 [95% CI, 1.160–4.763], *p* = 0.018) were associated with worse PFS. For the CR/PR group, lower hemoglobin concentration (Crude OR, 0.977 [95% CI, 0.961–0.994], *p =* 0. 0.007), lower platelet count (Crude OR, 1.004 [95% CI, 1.001–1.008], *p =* 0. 0.021), larger tumor size before NACT (Crude OR, 1.372 [95% CI, 1.042–1.808], *p =* 0.024) or after NACT (Crude OR, 1.880 [95% CI, 1.333–2.651], *p* < 0.001), > 1/2 depth of cervical invasion (Crude OR, 3.375 [95% CI, 1.624–7.017], *p =* 0.001), uterus involvement (Crude OR, 3.448 [95% CI, 1.201–9.897], *p =* 0.021), and lymph node metastasis (Crude OR, 3.982 [95% CI, 1.822–8.700], *p =* 0.001) were associated with worse PFS. For the SD/PD group, the clinicopathological risk factor for PFS was > 1/2 depth of cervical invasion (Crude OR, 3.303 [95% CI, 1.011–10.791], *p =* 0.048). When the candidate variables with a *p*-value of < 0.2 in univariate analysis of Cox proportional hazards regression were included in the multivariable model, deep cervical invasion was the only significant independent prognostic factor related to poor PFS for all patients undergoing NACT (Adjusted OR, 2.580 [95% CI, 1.310–5.079], *p =* 0.006). Additionally, deep cervical invasion was associated with poorer PFS in responders (Adjust. OR, 2.358 [95% CI, 1.092–5.090], *p* = 0.029). In addition, patients with lower hemoglobin concentrations (Adjusted OR, 0.976 [95% CI, 0.957-0.996], *p =* 0.020), larger tumor size after NACT (Adjust. OR 1.720 [95% CI, 1.085–2.726], *p =* 0.021), and lymph node metastasis (Adjusted OR, 2.391 [95% CI, 1.038-5.508], *p =* 0.041) had significantly worse PFS in responders. Nevertheless, when regarding non-responders to NACT, no clinical or pathological factors were associated with PFS (Table 1).

**Table 1:** Multivariate analysis of progression-free survival for patients undergoing NACT

	CR/PR (***n =*** 227)		SD/PD (***n =*** 91)		NACT (***n =*** 318)	
	Adjust ***p***-Value	Adjust OR (95%CI)	Adjust p-Value	Adjust OR (95%CI)	Adjust p-Value	Adjust OR (95%CI)
**Age**	\	\	0.308	0.967 (0.906, 1.032)	\	\
**Hb**	0.020	0.976 (0.957, 0.996)	\	\	0.121	0.987 (0.972, 1.003)
**Plt**	0.459	1.002 (0.997, 1.006)	\	\	0.357	1.002 (0.998, 1.005)
**BMI**	\	\	0.201	0.892 (0.749, 1.063)	0.568	0.971 (0.877, 1.075)
**Tumor size**						
**before NACT**	0.566	0.904 (0.642, 1.274)	\	\	0.923	0.987 (0.760, 1.282)
**after NACT**	0.021	1.720 (1.085, 2.726)	\	\	0.281	1.119 (0.912, 1.372)
**> 1/2 depth of cervical invasion**	0.029	2.358 (1.092, 5.090)	0.108	2.751 (0.803, 9.180)	0.006	2.580 (1.310, 5.079)
**LVSI**	\	\	\	\	0.857	0.927 (0.409, 2.102)
**Vagina involvement**	\	\	\	\	0.679	1.393 (0.290, 6.690)
**Uterus involvement**	0.233	1.998 (0.641, 6.225)	\	\	0.627	1.306 (0.445, 3.838)
**Lymph node metastasis**	0.041	2.391 (1.038, 5.508)	\	\	0.310	1.478 (0.696, 3.140)

Candidate variables with a p-value < 0.2 on univariate analysis of Cox proportional hazards regression were included in the multivariable model and “\” meant the corresponding factor was not included

Abbreviations: *OR* Odds ratio, *Hb* Hemoglobin concentration, *Plt* Platelet concentration, *BMI* Body mass index, *SCCA* Squamous Cell Carcinoma Antigen, *NACT* Neoadjuvant chemotherapy, *LVSI* lymphovascular space invasions

Next, we performed a univariate analysis affecting OS (Additional file 1: Table S6). For all patients undergoing NACT, the risk factors of OS were consistent with PFS, including lower hemoglobin concentrations (Crude OR, 0.979 [95% CI, 0.961–0.998], *p =* 0.029), larger tumor size before NACT (Crude OR, 1.456 [95% CI, 1.131–1.874], *p* = 0.004) or after NACT (Crude OR, 1.386 [95% CI, 1.140–1.685], *p =* 0.001), > 1/2 depth of cervical invasion (Crude OR, 3.832 [95% CI, 1.683–8.962], *p* = 0.002), and vagina involvement (Crude OR, 5.567 [95% CI, 1.303–23.790], *p =* 0.021). For responders, lower hemoglobin concentrations (Crude OR, 0.976 [95% CI, 0.953–0.999], *p =* 0.039), larger tumor size before NACT (Crude OR, 1.509 [95% CI, 1.080–2.106], *p =* 0.016) or after NACT (Crude OR, 1.945 [95% CI, 1.298–2.914], *p* = 0.001), > 1/2 depth of cervical invasion (Crude OR, 3.575 [95% CI, 1.299–9.840], *p =* 0.014), and lymph node metastasis (Crude OR, 3.785 [95% CI, 1.313–10.906], *p =* 0.014) were associated with worse OS. No factor was found resulting in worse OS for non-responders. However, when we analyzed the correlation between OS and factors by multivariate analysis, besides deep cervical invasion was associated with worse OS for all patients undergoing NACT (Adjusted OR, 2.608 [95% CI, 1.017–6.691], *p =* 0.046), no other clinicopathological factors were found associated with OS in multivariate regression, whether it was for responders, non-responders, or all patients undergoing NACT (Table 2).

**Table 2:** Multivariate analysis of overall survival for patients undergoing NACT

	CR/PR (***n =*** 227)		SD/PD (***n =*** 91)		NACT (***n =*** 318)	
	Adjust p-Value	Adjust OR (95%CI)	Adjust p-Value	Adjust OR (95%CI)	Adjust p-Value	Adjust OR (95%CI)
**Age**	\	\	0.108	0.925 (0.841, 1.017)	\	\
**Hb**	0.093	0.978 (0.953, 1.004)	\	\	0.300	0.989 (0.968, 1.010)
**Tumor size**						
**before NACT**	0.760	0.926 (0.563, 1.522)	0.945	1.029 (0.457, 2.319)	0.650	1.082 (0.771, 1.518)
**after NACT**	0.055	1.928 (0.987, 3.769)	0.463	1.306 (0.641, 2.660)	0.096	1.246 (0.962, 1.614)
**Approach of surgery**	\	\	\	\	0.085	0.305 (0.079, 1.176)
**FIGO**	0.272	1.920 (0.599, 6.152)	\	\	\	\
**> 1/2 depth of cervical invasion**	0.154	2.243 (0.739, 6.806)	0.325	2.357 (0.428, 12.97)	0.046	2.608 (1.017, 6.691)
**LVSI**	\	\	\	\	0.242	1.901 (0.648, 5.577)
**Vagina involvement**	0.523	0.427 (0.031, 5.831)	0.165	6.154 (0.472, 80.171)	0.676	1.476 (0.238, 9.157)
**Uterus involvement**	\	\	\	\	0.927	1.076 (0.227, 5.109)
**Lymph node metastasis**	0.464	1.591 (0.46, 5.507)	\	\	0.938	1.042 (0.368, 2.946)

Candidate variables with a p-value of < 0.2 in univariate analysis of Cox proportional hazards regression were included in the multivariable model and “\” meant the corresponding factor was not included

Abbreviations: *OR* Odds ratio, *Hb* Hemoglobin concentration, *Plt* Platelet concentration, *BMI* Body mass index, *SCCA* Squamous Cell Carcinoma Antigen, *NACT* Neoadjuvant chemotherapy, *LVSI* Lymphovascular space invasion

### Overview of post-NACT therapy for non-responders

In the 28 different centers, the treatment of responders was performed according to the experimental design, while the treatment of the non-responders varied according to the experience of attending physicians. Previous analyses have shown that patients who were not sensitive to NACT also achieved survival outcomes that were not inferior to those of NACT-sensitive patients, and we consider that the prognosis here may be related to a different choice of treatment regimen. Therefore, we focused our analysis on the treatment regimens for non-responders and explored whether and how the treatment affected the prognosis of non-responders.

**Fig. 4:**
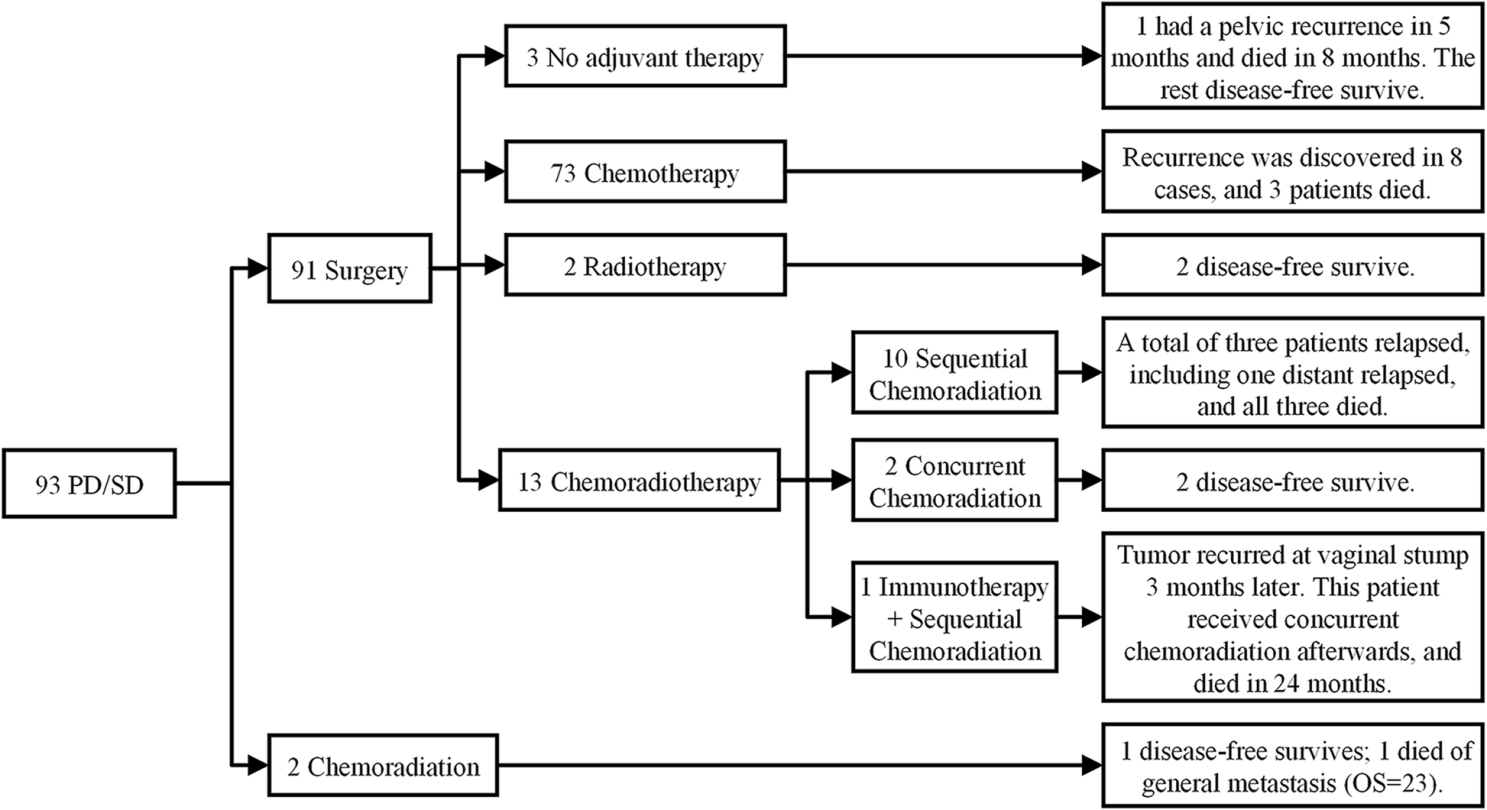
Therapy and Outcomes of Patients Insensitive to NACT. Abbreviations: SD, stable disease; PD, progressive disease; OS, overall survival

Detailed information on the treatment and survival outcome of 93 patients insensitive to NACT was given in Fig. 4 and Table 3. Of these patients, 91 of 93 (97.8%) non-responders received RS plus pelvic lymphadenectomy following NACT, and only two patients received sequential chemoradiation (SCRT) after NACT. For two SCRT patients, tumor general metastasis to the cervix, segment VIII of the liver, and the middle and lower lobes of left lung were identified in one patient (PFS = 15), and she died 23 months after entering the experiment (P276 in Table 3); another patient survived disease-free throughout the follow-up period.

**Table 3:** Characteristics and treatment details of patients insensitive to NACT who died or developed recurrent disease

Patient No.	Age	FIGO stage	Histology	Deep cervical invasion	LVSI	Lymph node status	Risk level	Therapy after surgery	Patterns of disease recurrence	Therapy after recurrence	Time to recurrence (months)	Time to death (months)
P366	47	IB3	SCC	Positive	Negative	Negative	Intermediate	Chemoradiation	Pelvic Cavity	Untreated	4	6
P294	47	IB3	SCC	Positive	Positive	Negative	Intermediate	None	Pelvic Cavity	Untreated	5	8
P133	44	IIA2	SCC	Negative	Negative	Negative	Low	Chemotherapy	Unknown	Unknown	Unknown	12
P137	42	IIA2	SCC	Negative	Negative	Negative	Low	Chemotherapy	Sigmoid Colon	Radiotherapy	9	16
P080	44	IIA2	SCC	Positive	Negative	Negative	Intermediate	Immu+ Chemoradiotherapy	Cervical Stump	Chemoradiation	3	24
P143	51	IB3	SCC	Positive	Negative	Negative	Intermediate	Chemotherapy	Unknown	Unknown	7	25
P219	32	IB3	SCC	Positive	Positive	Negative	Intermediate	Chemoradiation	Uterine Cervix	Radiotherapy	29	29
P053	34	IIA2	SCC	Positive	Negative	Positive	High	Chemoradiation	Retroperitoneal Lymph Node	Chemoradiation	19	45
P241	47	IB3	SCC	Positive	Negative	Negative	Low	Chemotherapy	Bladder	Radiotherapy	7	35, Alive
P167	43	IIA2	SCC	Negative	Negative	Negative	Low	Chemotherapy	Cervical Stump	Reoperation+Immu.	19	52, Alive
P121	39	IIA2	SCC	Positive	Positive	Negative	Intermediate	Chemotherapy	Cervical Stump	Radiotherapy	20	39, Alive
P373	54	IB3	SCC	Positive	Negative	Negative	Low	Chemotherapy	Pelvic Cavity	Radiotherapy	24	59, Alive
P292	46	IIA2	SCC	Negative	Negative	Negative	Low	Chemotherapy	para-aortic lymph nodes	NA	36	36, Alive
P276	46	IIA2	SCC	NA	NA	NA	NA	NA	Cervix, liver, and lung	Unknown	15	23

Patient No.276 received concurrent chemoradiotherapy after neoadjuvant chemotherapy, and had no surgically pathological results. For Patient 292, recurrence was found in the last follow-up (2021–11), so the treatment options and long-term prognosis after recurrence were not available at the time of writing

Abbreviations: *FIGO* International Federation of Gynecology and Obstetrics, *LVSI* Lymph vascular space invasion, *SCC* Squamous cell carcinoma, *AC* Adenocarcinoma, *NA* Not available, *Immu* Immunotherapy

Detailed operation information on patients undergoing NACT is shown (Additional file 1: Table S7). Since the result of the LACC Trial [26] was not available at the time of the implementation of this clinical trial, the majority of patients underwent a minimally invasive hysterectomy (228 cases, 74.5%). In SD/PD group, the open surgery rates were 31.8%, which was slightly higher than that in CR/PR group (23.1%; Chi-square test, *p =* 0.143). It may result from the difficulty of SD/PD patients’ surgery. During the operation, intraoperative hemorrhage (*p =* 0.302) and length of parametrium excision (*p =* 0.233) for the two groups showed no significant differences. Operative complications were recorded as well and a total of 23 patients (7.2%) suffered from at least one kind of operative complication including injury to the urinary system, injury to the gastrointestinal tract, vascular injury, infection, deep vein thrombosis, and lymphocyst with a diameter more than five centimeters. The rate of any operative complications at the time of the analysis was 7.0% in the CR/PR group and 7.7% in the SD/PD group. After surgery, the median indwelling catheter time in the CR/PR group (5 days) was shorter than that in SD/PD group (6 days; Chi-square test, *p =* 0.033), and the median amount of post-operation drainage in the CR/PR group (390 mL) was less than that in the SD/PD group (550 mL; Chi-square test, *p =* 0.060).

In 91 patients receiving radical hysterectomy following NACT, three patients did not receive any postoperative adjuvant therapy, two of whom survived throughout the follow-up period. Patient No.294 refused any treatment after surgery and even after recurrence and died 8 months after being enrolled in the clinical trial. The vast majority of patients (80.2%) received adjuvant chemotherapy, and their chemotherapy regimen was consistent with NACT. Among them, eight patients relapsed (P121, P133, P137, P143, P167, P241, P292, P373 in Table 3) and three patients died. A total of 15 patients underwent radiotherapy with (2 patients) or without (13 patients) platinum-containing chemotherapy or immunotherapy as adjuvant therapy. Among them, recurrence was observed in four patients and they all died from disease-specific causes (P366, P080, P219, P053). Of the five patients that survived recurrence, three received radiotherapy after recurrence, one had a reoperation, and one patients’ treatment regimen was not available.

### Prognostic significance of therapeutic factors for SD/PD group

**Fig. 5:**
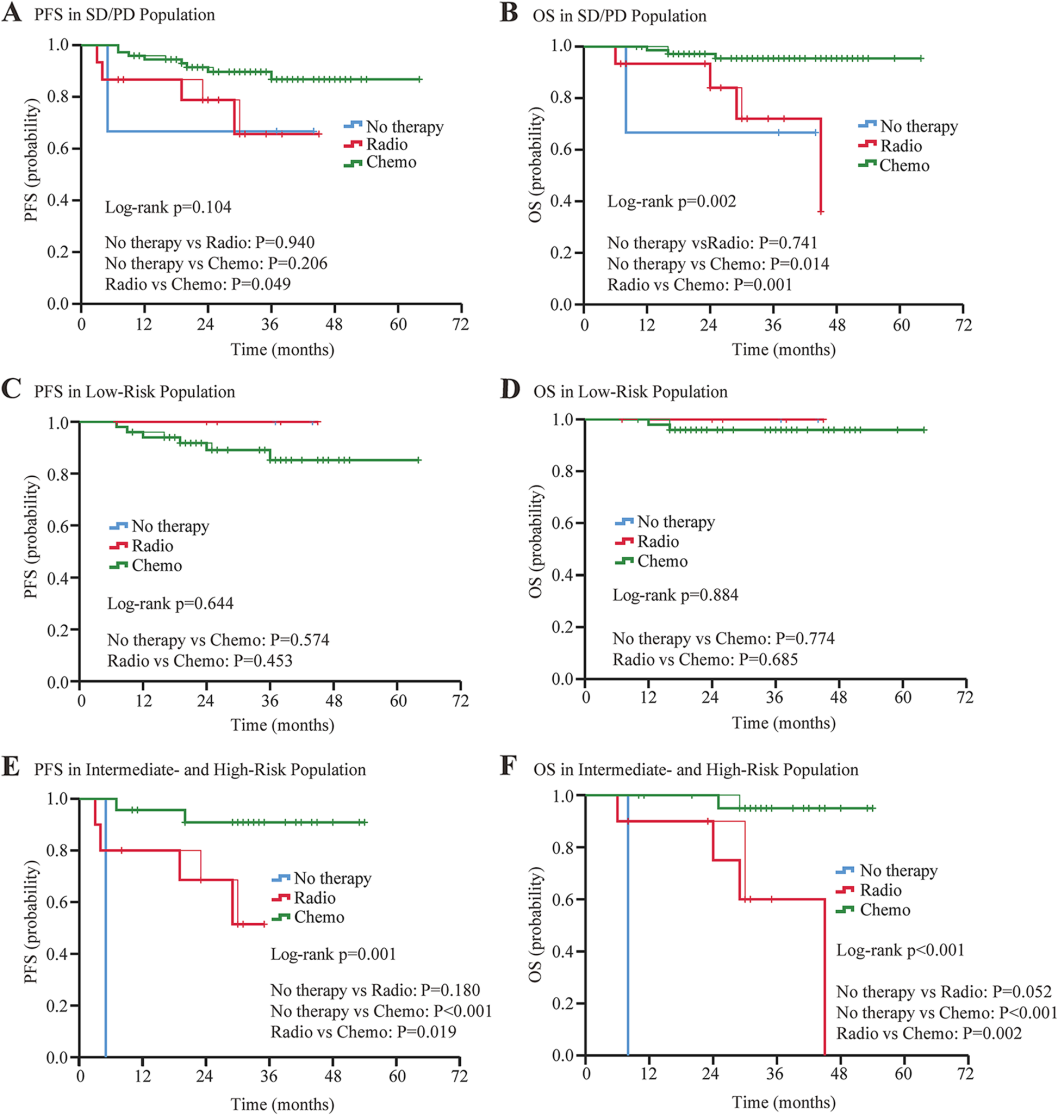
Progression Free Survival and Overall Survival Curves for Patients Insensitive to NACT. Figure 5 included 91 patients receiving neoadjuvant chemotherapy followed by radical surgery. Log-rank test was used to test the difference in PFS and OS of three interventions, and interventions were compared in pairs as well. Kaplan–Meier plot showed progression free survival and overall survival of all SD/PD population (**A** and **B**), of patients in low-risk group (**C** and **D**) and in intermediate- and high-risk group (**E** and **F**). Tick marks indicate censored data. Abbreviations: PFS, progression free survival; OS, overall survival; CR, clinical response; PR, partial response; SD, stable disease; PD, progressive disease

To better distinguish the effect of different adjuvant therapies on the prognosis of the 91 NACT-insensitive patients who received NACT followed by RS, we performed Kaplan–Meier survival analysis and log-rank test and found that only the chemotherapy group had a significantly better PFS and OS than the radiotherapy group (PFS, *p* = 0.049; OS, *p =* 0.001) (Fig. 5A and B). Considering that different pathological results would affect the choice of treatment, patients were divided into two groups based on risk according to the Sedlis criteria: (1) low-risk and (2) intermediate and high risk group using recognized high-risk pathologic features after radical hysterectomy [27].

In the low-risk group (*n =* 57), two patients did not receive any adjuvant therapy, two patients received only radiotherapy, and three patients received sequential chemoradiation. None of the above seven patients had cancer progression or died. The remaining 50 patients received only chemotherapy as adjuvant therapy, and among them six patients had died or had disease progression (Table 3). Based on this, we found that adjuvant treatment was not a significant independent prognostic factor for PFS (*p =* 0.644; no adjuvant treatment vs. chemotherapy alone, *p =* 0.574; chemotherapy alone vs. radiotherapy, *p =* 0.453) or OS (*p =* 0.884; no adjuvant treatment vs. chemotherapy alone, *p =* 0.774; chemotherapy alone vs. radiotherapy, *p =* 0.685) in the low-risk group (Fig. 5C and D), indicating that additional postoperative treatment did not help to improve the prognosis of patients in the low-risk group.

Among the 34 patients in the intermediate or high-risk group, 23 patients received only chemotherapy, seven patients received sequential chemoradiation, two patients received concurrent chemoradiation, one patient received immunotherapy plus chemoradiotherapy, and one patient refused any adjuvant therapy. Detailed information of death or recurrence is given in Table 3. Postoperative treatment appeared to be beneficial for patients in the intermediate- and high-risk group. We found that the use of chemotherapy decreased disease progression and increased survival compared with radiotherapy (PFS, *p =* 0.019; OS, *p =* 0.002) (Fig. 5E and F).

## Discussion

Our study showed that there was no significant difference in prognosis between NACT group and PST group in patients with stage IB3 and IIA2 cervical cancer in 28-centers from January 2016 to October 2019, and sensitivity to NACT does not affect clinical outcomes. It was also found that gynecologic oncologists in China prefer surgery for NACT-insensitive patients and that postoperative adjuvant chemotherapy has a significant survival advantage over other treatments for patients with intermediate- or high- risk factors. This finding suggests attention to the possible shift in sensitivity to chemotherapy after tumor load reduction and provides new ideas for postoperative treatment of patients with NACT-insensitive cervical cancer.

The use of NACT for locally advanced cervical cancer is controversial, mainly focusing on whether NACT can improve the prognosis of patients with locally advanced cervical cancer. A phase III study (JCOG 0102) in Japan found that NACT+RS did not achieve better OS compared with PST in patients with stages IB2, IIA2, and IIB locally advanced cervical cancer [22]. An international collaborative meta-analysis included 5 RCTs and 4 observational studies involving 1784 patients among 523 potentially relevant studies between January 1987 and September 2010 also did not find that NACT led to better OS [28]. However another meta-analysis of 6 randomized trials including patients with early or locally advanced cervical cancer found that patients who underwent NACT plus radical hysterectomy had better PFS and OS compared to those who underwent a primary radical hysterectomy, regardless of total CDDP dose, chemotherapy cycle length or tumor stage [15]. Additionally, NACT plus RS showed the more obvious effect of eliminating positive lymph nodes to be a valuable and reasonable treatment option in patients with stage IB1-IIB cervical cancer [29]. Our study from the 28-different centers identified an OS of 85.8% and a PFS of 80.5% for the NACT group, while OS was 87.8% and PFS was 83.8% for the PST group. This suggests that the NACT group did not present have a better prognosis than the PST group.

Past studies have shown that that platinum-based NACT followed by RS in locally advanced cervical cancer had 5-year DFS rates ranging from 55.4 to 71% and 5-year OS rates ranging from 58.9 to 81%, respectively [17, 20, 22, 30–37]. The results of our experiment are consistent with the literature but with slightly higher rates. This may be related to the inclusion of patients with more advanced staging in other studies as well as more adequate postoperative adjuvant chemotherapy in our study. It should also be reminded that although our study was a randomized controlled clinical trial and the two groups were not completely balanced at baseline, especially in terms of tumor size and FIGO stage, which may also be an important factor affecting the efficacy of the NACT group.

In addition, 71.4% of patients who received NACT experienced an objective response, while 38.6% showed resistance to NACT. Studies found that 55.6–77.6% of the NACT-treated patients got CR/PR, which is consistent with our conclusions in this multicenter clinical study [38–40]. Meanwhile, we surprisingly found that there was no difference in PFS and OS in patients with stage IB3 and IIA2 disease who were insensitive to NACT as compared with patients sensitive to NACT in this multicenter retrospective study. However, in past studies, chemotherapy sensitivity has always been an important factor affecting the prognosis of patients [16, 17, 41]. Some studies hypothesize that patients who did not obtain an overall optimal response unnecessarily delay effective local therapy, thus leading to a higher risk of recurrence and a higher risk of death than those who obtained an overall optimal response [17, 23, 35, 42–46] Similar survival outcomes in the CR/PR and SD/PD groups in this multicenter clinical study may be related to the subsequent treatment modality of NACT-insensitive patients. Although the treatment of SD/PD patients was not specified in our study, most of the attending physicians in general hospitals tend to adopt diversified and individualized treatments based on experience, and choose surgery plus postoperative adjuvant therapy, which may be the main reason for the similar OS and PFS of SD/PD patients to those in CR/PR group [40, 47].

We then further analyzed the prognostic factors and found that in patients receiving NACT, those sensitive to NACT and > 1/2 depth of cervical invasion were high-risk factors for poor PFS. In patients sensitive to NACT, lymph node metastasis is another risk factor for poor PFS. Similar to what other investigators have found, lymph node invasion and deep myometrial invasion are very important indicators of prognosis in cervical cancer [48–52]. Besides, we further clarified that smaller tumor size after NACT was correlated to better PFS only for patients sensitive to NACT, as smaller tumor sizes represented favorable prognostic variables of patients treated with this chemo-surgical approach [17, 30, 32].

Past studies have indicated a correlation between hemoglobin values and survival outcomes in cancer patients [53], and our study also found that in the CR/PR group patients with lower hemoglobin concentration before treatment were associated with a worse PFS. Preoperative anemia may be associated with tumor bleeding, and the main factor contributing to tumor bleeding is tumor aggressiveness to surrounding tissues which may imply that tumor aggressiveness in CR/PR patients may be associated with PFS. Deep cervical invasion may be a factor affecting OS, but when analyzing NACT-insensitive patients alone, we were surprised to find that only subsequent treatment modalities could affect the prognosis of these patients.

Given that the prognosis of NACT-insensitive patients was found to correlate with postoperative treatment, we then analyzed the effect of different postoperative adjuvant therapies on prognosis, and found that in comparison with therapy containing radiotherapy, adjuvant chemotherapy was associated with improved OS, but not PFS. Additionally, the prognosis of patients not responsive to NACT comparable to that of responders after individualized treatment. We further found that the non-responders with low pathological risk factors had similar PFS and OS, regardless of what adjuvant treatment was given. Thus, patients with intermediate or high pathological risk factors could benefit from adjuvant chemotherapy. As shown in Table 3, five of the 14 patients who relapsed and survived, were all postoperative chemotherapy patients. On the choice of treatment after relapse in these six patients, one underwent secondary surgery, and three underwent radiotherapy, which showed that postoperative chemotherapy was superior in the choice of treatment following recurrence. One patient was suspected of recurrence at the last follow-up visit, and her treatment after recurrence and future survival were not available at the time of writing. It is well known that patients who have not had radiotherapy can undergo either surgery or high-intensity radiotherapy after recurrence. However, these treatments are very risky for patients who have already received full-dose radiotherapy.

Among the nine patients who died after recurrence, four patients received chemoradiotherapy after surgery, and three patients received chemotherapy after surgery. This also confirmed from that concurrent chemoradiotherapy did not improve survival compared with chemotherapy and may be related to the limited treatment options available for patients who have relapsed after concurrent chemoradiotherapy. Our findings suggest that NACT insensitivity does not affect the choice of chemotherapy after the operation. This may be related to the reduction in tumor load after the resistant lesion is removed, allowing the patient to regain sensitivity to chemotherapy.

Our study has several limitations. Firstly, although it is a prospective randomized controlled trial, there was some bias in the enrollment process. Secondly, because the population analyzed in this paper is the NACT group, which is a subgroup study in the overall study, its level of evidence is lower than prospective randomized controlled trials. Thus, future clinical trials with larger sample sizes and more rigorous designs are still required to reach more convincing conclusions.

## Conclusions

This multicenter clinical study found that for patients with FIGO stage cervical cancer IB3 and IIA2, NACT + RS therapy is not inferior to direct surgery, and sensitivity to NACT is not a factor affecting the prognosis of non-responders, while follow-up adjuvant therapy is a factor. Additionally, among patients who are insensitive to NACT, it is proposed for the first time that for patients at an intermediate or a high risk, continuing chemotherapy after surgery may be a treatment option with good prognosis and clinical application prospects. In a follow-up study, we will further conduct a prospective randomized controlled study with a higher level of evidence for this group of patients to provide a clinical basis for more effective treatment methods for this group of patients with refractory locally advanced cervical cancer.

## Supplementary Information

**Additional file 1.**

## Data Availability

The datasets supporting the conclusions of this article are included within the article and its additional file, further inquiries can be directed to the corresponding author.

## Abbreviations

BMI: Body mass index
CCRT: Concurrent platinum-based chemoradiation
CI: Confidence intervals
CR: Clinical response
DSS: Disease-specific survival
FIGO: International Federation of Gynecology and Obstetrics
HR: Hazard ratio
ITT: Intention-to-treat
NACT: Neoadjuvant chemotherapy
ORs: Odds ratios
OS: Overall survival
PD: Progressive disease
PFS: Progression free survival
OS: Overall survival
PP: Per-protocol
PR: Partial response
PST: Primary surgery treatment
RECIST: Response Evaluation Criteria in Solid Tumors
RS: Radical surgery
SD: Stable disease
