# Supplementary material for: Neoadjuvant chemotherapy for patients with international federation of gynecology and obstetrics stages IB3 and IIA2 cervical cancer: a multicenter prospective trial
Source: BMC Cancer. 2022 Dec 5;22:1270. doi: 10.1186/s12885-022-10355-3 (PMC9724322; doi:10.1186/s12885-022-10355-3)
Supplement: Supplementary file 1 — Additional file 1. [file 12885_2022_10355_MOESM1_ESM.docx]

**Neoadjuvant Chemotherapy for Patients with International Federation of Gynecology and Obstetrics Stages IB3 and IIA2 Cervical Cancer: A Multicenter Prospective Trial**

**Additional file 1**

## **Table S1. Characteristics of Patients at Baseline**

| **Characteristic** | **NACT (n=369)** | **PST (n=368)** | **p-Value** |
| --- | --- | --- | --- |
| **Median age, year (range)** | 48 (20, 65) | 48 (22, 65) | 0.290 |
| **Median hemoglobin concentration, g/L (range)** | 122 (56, 162) | 124 (50, 233) | 0.163 |
| **Median platelet concentration, *10^9^/L (range)** | 253 (52, 703) | 243 (82, 546) | 0.051 |
| **Median body mass index, kg/m^2^ (range)** | 22.8 (15.8, 36.3) | 22.7 (15.2, 34.4) | 0.456 |
| **Median tumor size, cm (range)** | 4.0 (0.5, 9.6) | 4.0 (0.6, 7.2) | 0.005 |
| **FIGO stage —no. (%)** |  |  | <0.001 |
| **IB3** | 138 (37.4) | 204 (55.4) |  |
| **IIA2** | 231 (62.6) | 164 (44.6) |  |

All data were obtained before receiving any treatment for cervical cancer. p-values were derived from Mann–Whitney U tests for continuous variables and two-sided Fisher’s exact test for the categorical variables. The baseline clinical and demographic characteristics were not well balanced. The tumor size in the greatest dimension in the NACT group was larger than that in the PST group, and the rate of FIGO stage IIA2 was higher in the NACT group than in the PST group.

**Table S2.** **Baseline Characteristics of Patients Undergoing NACT**

| **Characteristic** | **CR/PR (n=227)** | **SD/PD (n=93)** | **p-Value** |
| --- | --- | --- | --- |
| **Median age, years (range)** | 49 (20, 63) | 47 (28, 64) | 0.216 |
| **Median** **hemoglobin concentration, g/L (range)** | 122 (56, 162) | 122 (64, 151) | 0.310 |
| **Median** **platelet concentration, *10^9^/L (range)** | 251 (52, 703) | 252 (117, 593) | 0.738 |
| **Median** **body mass index, kg/m^2^ (range)** | 22.8 (17.6, 34.5) | 22.6 (15.8, 33.2) | 0.573 |
| **Median tumor size, cm (range)** | 4.1 (0.5, 8.7) | 4.1 (1.7, 9.6) | 0.953 |
| **FIGO stage, No. (%)** |  |  | 0.529 |
| **IB3** | 84 (37.0) | 38 (40.9) |  |
| **IIA2** | 143 (63.0) | 55 (59.1) |  |

No clinical and demographic difference was noticed between the CR/PR group and the SD/PD group.

## **Table S3. Postoperative Histopathologic Characteristics^a^**

| **Pathological Manifestations** | **CR/PR (n=227)** | **SD/PD (n=91)** | **P-value^b^** |
| --- | --- | --- | --- |
| **Pathological type, No. (%)** |  |  | 0.454 |
| **Squamous cell carcinoma** | 204 (89.9) | 84 (92.3) |  |
| **Adenocarcinoma** | 18 (7.9) | 4 (4.4) |  |
| **Squamous adenocarcinoma** | 5 (2.2) | 3 (3.3) |  |
| **>1/2 depth of cervical invasion, n (%)** | 77 (33.9) | 40 (44.0) | 0.097 |
| **Positive vaginal resection margin** | 1 (0.4) | 0 (0) | 1.000 |
| **LVSI, n (%)** | 28 (12.3) | 14 (15.4) | 0.468 |
| **Parametrial infiltration, n (%)** | 2 (0.9) | 3 (3.3) | 0.144 |
| **Vagina involvement, n (%)** | 4 (1.8) | 3 (3.3) | 0.413 |
| **Uterus involvement, n (%)** | 11 (4.8) | 7 (7.7) | 0.420 |
| **Lymph node metastasis, n (%)** | 24 (10.6) | 14 (15.4) | 0.253 |

All patients in the CR/PR group and 91 of 93 patients in the SD/PD group received surgery after neoadjuvant therapy. p-values were derived from Fisher’s exact test.

Abbreviations: LVSI, lymphovascular space invasion.

**Table S4. Recurrence and Death of Patients Undergoing NACT**

| **Recurrence or Death** | **CR/PR (n=227)** | **SD/PD (n=93)** |
| --- | --- | --- |
| **Recurrence, n(%)** | 25 (11.0) | 13 (14.0) |
| **Local recurrence^a^** | 19 (8.4) | 9 (9.7) |
| **Distant recurrence^b^** | 4 (1.8) | 3 (3.2) |
| **Unknown** | 2 (0.9) | 1 (1.1) |
| **Death from tumor, n (%)** | 14 (6.2) | 9 (9.7) |
| **Death from others, n (%)^c^** | 2 (0.9) | 0 (0) |

^a^Local recurrence included tumor and lymph metastasis located in the pelvis.

^b^Distant recurrence included distant metastasis beyond pelvic and lymph metastasis in the para-aortic area.

^c^In total, two patients died from other causes, including one from suicide and one from COVID-19.

**Table S5**. **Univariate Analysis of Factors Influencing Progression-free Survival in Patients Undergoing NACT^a^**

|  | **CR/PR (n=227)** | |  | **SD/PD (n=91)** | |  | **NACT (n=318)** | |
| --- | --- | --- | --- | --- | --- | --- | --- | --- |
|  | **Crude p-Value** | **Crude OR (95%CI)** |  | **Crude p-Value** | **Crude OR (95%CI)** |  | **Crude p-Value** | **Crude OR (95%CI)** |
| **Age** | 0.958 | 0.999 (0.952, 1.047) |  | 0.149 | 0.955 (0.898, 1.017) |  | 0.356 | 0.983 (0.948, 1.020) |
| **Hb** | 0.007 | 0.977 (0.961, 0.994) |  | 0.344 | 0.988 (0.963, 1.013) |  | 0.005 | 0.980 (0.966, 0.994) |
| **Plt** | 0.021 | 1.004 (1.001, 1.008) |  | 0.727 | 0.999 (0.992, 1.005) |  | 0.096 | 1.003 (1.000, 1.006) |
| **BMI** | 0.685 | 0.975 (0.864, 1.101) |  | 0.081 | 0.85 (0.708, 1.020) |  | 0.180 | 0.932 (0.842, 1.033) |
| **Change of tumor size** | 0.449 | 1.115 (0.841, 1.480) |  | 0.620 | 1.127 (0.701, 1.812) |  | 0.347 | 1.099 (0.903, 1.336) |
| **Tumor size** |  |  |  |  |  |  |  |  |
| **before NACT** | 0.024 | 1.372 (1.042, 1.808) |  | 0.639 | 1.090 (0.759, 1.566) |  | 0.039 | 1.253 (1.011, 1.552) |
| **after NACT** | <0.001 | 1.880 (1.333, 2.651) |  | 0.451 | 1.134 (0.818, 1.572) |  | 0.007 | 1.255 (1.064, 1.481) |
| **Approach of surgery^b^** | 0.498 | 0.717 (0.274, 1.876) |  | 0.829 | 0.878 (0.270, 2.855) |  | 0.530 | 0.790 (0.379, 1.647) |
| **FIGO^c^** | 0.283 | 1.536 (0.701, 3.365) |  | 0.813 | 0.880 (0.305, 2.537) |  | 0.464 | 1.263 (0.676, 2.358) |
| **Pathological type** | 0.986 | NA |  | 0.748 | NA |  | 0.811 | NA |
| **>1/2 depth of cervical invasion** | 0.001 | 3.375 (1.624, 7.017) |  | 0.048 | 3.303 (1.011, 10.791) |  | <0.001 | 3.359 (1.808, 6.243) |
| **Positive vaginal resection margin** | 0.817 | 0.049 (0, 5.86E+9) |  | NA | NA |  | 0.813 | 0.050 (0, 3.25E+9) |
| **LVSI** | 0.375 | 1.545 (0.590, 4.043) |  | 0.337 | 1.886 (0.516, 6.893) |  | 0.192 | 1.669 (0.773, 3.603) |
| **Parametrial infiltration** | 0.720 | 0.049 (0, 724338.8) |  | 0.648 | 0.047 (0, 22959.7) |  | 0.569 | 0.048 (0, 1604.003) |
| **Vagina involvement** | 0.339 | 2.649 (0.359, 19.532) |  | 0.223 | 3.577 (0.460, 27.794) |  | 0.123 | 3.062 (0.738, 12.698) |
| **Uterus involvement** | 0.021 | 3.448 (1.201, 9.897) |  | 0.995 | 1.007 (0.131, 7.748) |  | 0.071 | 2.362 (0.929, 6.002) |
| **Lymph node metastasis** | 0.001 | 3.982 (1.822, 8.700) |  | 0.429 | 0.439 (0.057, 3.370) |  | 0.018 | 2.350 (1.160, 4.763) |

^a^p-values were derived from Cox proportional hazards regression analysis.

^b^OR represented the odds of the event in patients receiving open abdominal radical hysterectomy versus patients receiving laparoscopic or robot-assisted radical hysterectomy.

^c^OR represented the odds of the event in patients with FIGO stage IIA2 versus in patients with FIGO stage IB3.

Abbreviations: OR, odds ratio; Hb, hemoglobin concentration; Plt, platelet count; BMI, body mass index; NACT, neoadjuvant chemotherapy; FIGO, International Federation of Gynecology and Obstetrics; LVSI, lymphovascular space invasion; NA, Not applicable.

**Table S6**. **Univariate Analysis of Factors Influencing Overall Survival in Patients Undergoing NACT^a^**

|  | **CR/PR (n=227)** | |  | **SD/PD (n=91)** | |  | **NACT (n=318)** | |
| --- | --- | --- | --- | --- | --- | --- | --- | --- |
|  | **Crude p-Value** | **Crude OR (95%CI)** |  | **Crude p-Value** | **Crude OR (95%CI)** |  | **Crude p-Value** | **Crude OR (95%CI)** |
| **Age** | 0.820 | 1.008 (0.941, 1.079) |  | 0.153 | 0.944 (0.871, 1.022) |  | 0.420 | 0.980 (0.932, 1.030) |
| **Hb** | 0.039 | 0.976 (0.953, 0.999) |  | 0.423 | 0.987 (0.956, 1.019) |  | 0.029 | 0.979 (0.961, 0.998) |
| **Plt** | 0.350 | 1.002 (0.997, 1.007) |  | 0.397 | 1.003 (0.996, 1.01) |  | 0.248 | 1.002 (0.998, 1.006) |
| **BMI** | 0.801 | 0.979 (0.829, 1.156) |  | 0.211 | 0.866 (0.692, 1.085) |  | 0.340 | 0.936 (0.816, 1.073) |
| **Change of tumor size** | 0.827 | 1.045 (0.706, 1.547) |  | 0.537 | 1.218 (0.652, 2.278) |  | 0.464 | 1.104 (0.848, 1.437) |
| **Tumor size** |  |  |  |  |  |  |  |  |
| **before NACT** | 0.016 | 1.509 (1.080, 2.106) |  | 0.104 | 1.375 (0.937, 2.017) |  | 0.004 | 1.456 (1.131, 1.874) |
| **after NACT** | 0.001 | 1.945 (1.298, 2.914) |  | 0.053 | 1.415 (0.995, 2.011) |  | 0.001 | 1.386 (1.140, 1.685) |
| **Approach of surgery^b^** | 0.419 | 0.542 (0.123, 2.389) |  | 0.211 | 0.262 (0.032, 2.133) |  | 0.166 | 0.425 (0.127, 1.426) |
| **FIGO^c^** | 0.186 | 2.153 (0.691, 6.705) |  | 0.765 | 0.818 (0.22, 3.049) |  | 0.377 | 1.462 (0.630, 3.396) |
| **Pathological type** | 0.741 | NA |  | 0.816 | NA |  | 0.601 | NA |
| **>1/2 depth of cervical invasion** | 0.014 | 3.575 (1.299, 9.840) |  | 0.079 | 4.214 (0.844, 21.029) |  | 0.002 | 3.832 (1.638, 8.962) |
| **Positive vaginal resection margin** | 0.896 | 0.049 (0, 1.74E+18) |  | NA | NA |  | 0.882 | 0.050 (0, 1.00E+16) |
| **LVSI** | 0.255 | 2.079 (0.589, 7.331) |  | 0.293 | 2.414 (0.468, 12.46) |  | 0.112 | 2.234 (0.828, 6.026) |
| **Parametrial infiltration** | 0.807 | 0.049 (0, 1.54E+9) |  | 0.745 | 0.047 (0, 4723135) |  | 0.697 | 0.049 (0, 1.95E+5) |
| **Vagina involvement** | 0.189 | 3.883 (0.512, 29.460) |  | 0.060 | 7.679 (0.915, 64.448) |  | 0.021 | 5.567 (1.303, 23.790) |
| **Uterus involvement** | 0.241 | 2.432 (0.551, 10.734) |  | 0.621 | 1.697 (0.208, 13.829) |  | 0.181 | 2.284 (0.680, 7.669) |
| **Lymph node metastasis** | 0.014 | 3.785 (1.313, 10.906) |  | 0.753 | 0.716 (0.089, 5.738) |  | 0.056 | 2.452 (0.977, 6.154) |

^a^p-values were derived from Cox proportional hazards regression analysis.

^b^OR represented the odds of the event in patients receiving open abdominal radical hysterectomy versus patients receiving laparoscopic

or robot-assisted radical hysterectomy.

^c^OR represented the odds of the event in patients with FIGO stage IIA2 versus in in patients with FIGO stage IB3.

Abbreviations: OR, odds ratio; Hb, hemoglobin concentration; Plt, platelet count; BMI, body mass index; NACT, neoadjuvant chemotherapy; FIGO, International Federation of Gynecology and Obstetrics; LVSI, lymphovascular space invasion; NA, Not applicable.

**Table S7.** **Detailed Operation Information of Patients Undergoing NACT**

|  | **CR/PR (n=227)** | **SD/PD (n=91)** | **P-value^a^** |
| --- | --- | --- | --- |
| **Type of radical hysterectomy, n (%)** |  |  |  |
| **Laparotomy** | 51 (23.1) | 27 (31.8) | 0.143 |
| **Laparoscopy/Robot** | 170 (76.9) | 58 (68.2) |  |
| **Unknown** | 6 | 6 |  |
| **Median intraoperative hemorrhage, mL (range)** | 200 (30-1000) | 200 (20-1500) | 0.302 |
| **Median** **parametrium excision, cm (range)** | 3.0 (2.0-5.0) | 3.0 (2.5-5.0) | 0.233 |
| **Operative complication** |  |  |  |
| **Injury of urinary system, n (%)** | 0 (0) | 1 (1.1)^a^ |  |
| **Injury of gastrointestinal tract, n (%)** | 1 (0.4)^b^ | 0 (0) |  |
| **Injury of vessel, n (%)** | 0 (0) | 0 (0) |  |
| **Infection, n (%)^c^** | 2 (0.9) | 5 (5.5) |  |
| **Lymphocyst, n (%)^d^** | 13 (5.7) | 1 (1.1) |  |
| **Deep vein thrombosis, n (%)** | 1 (0.4) | 0 (0) |  |
| **Median catheter time, days (range)^e^** | 5 (0-23) | 6 (0-25) | 0.033 |
| **Median post-operation drainage, mL (range)** | 390 (0-4612) | 550 (0-8010) | 0.060 |

^a^The patient had a ureteral injury during the operation.

^b^The patient had a postoperative intestinal obstruction.

^c^Wound infection, pelvic infection, pulmonary infection, and other infections were all involved in statistical analysis.

^d^Lymphatic cysts with a diameter >5 cm were involved in statistical analysis.

^e^The indwelling catheter time was recorded as 0 days if the catheter was pulled out within 24 hours after the operation.

**Table S8. List of all participating centers**

| No. | Organization |
| --- | --- |
| 1 | Tongji Hospital, Tongji Medical College, Huazhong University of Science and Technology |
| 2 | Women's Hospital, School of Medicine, Zhejiang University |
| 3 | Hunan Cancer Hospital/The Affiliated Cancer Hospital of Xiangya School of Medicine, Central South University |
| 4 | The First Affiliated Hospital of Chongqing Medical University |
| 5 | Third Xiangya Hospital of Central South University |
| 6 | Renmin Hospital of Wuhan University |
| 7 | The First Affiliated Hospital of Nanjing Medical University |
| 8 | Cancer Hospital of China Medical University, Liaoning Cancer Hospital and Institute |
| 9 | Southwest Hospital, Third Military Medical University |
| 10 | Zhujiang Hospital, Southern Medical University |
| 11 | The Central Hospital of Wuhan, Tongji Medical College, Huazhong University of Science and Technology |
| 12 | Xiangyang Central Hospital, Affiliated Hospital of Hubei University of Arts and Science |
| 13 | Xiangya Hospital, Central South University |
| 14 | The First Peoples Hospital of Jingzhou |
| 15 | First Affiliated Hospital of Shenzhen University |
| 16 | The First Affiliated Hospital of Soochow University |
| 17 | The Second Hospital of Jilin University |
| 18 | First Affiliated Hospital of Xinjiang Medical University |
| 19 | Yunnan Cancer Hospital, The Third Affiliated Hospital of Kunming Medical University |
| 20 | Shenzhen Hospital of Beijing University |
| 21 | Jingzhou 2nd People's Hospital |
| 22 | The Second Affiliated Hospital of Wenzhou Medical University |
| 23 | Tianjin Central Hospital of Obstetrics and Gynecology |
| 24 | The Second Xiangya Hospital of Central South University |
| 25 | The Second Affiliated Hospital, Kunming Medical University |
| 26 | Shanghai General Hospital, Shanghai Jiao Tong University, School of Medicine |
| 27 | Affiliated Shenzhen Maternity and Child Healthcare Hospital, Southern Medical University |
| 28 | The First Affiliated Hospital of Medical College, Shihezi University |
